# The prevalence of pathogens in ticks collected from humans in Belgium, 2021, versus 2017

**DOI:** 10.1186/s13071-024-06427-x

**Published:** 2024-09-05

**Authors:** Camille Philippe, Laurence Geebelen, Marie R. G. Hermy, François E. Dufrasne, Katrien Tersago, Alessandro Pellegrino, Manoj Fonville, Hein Sprong, Marcella Mori, Tinne Lernout

**Affiliations:** 1 Sciensano, Belgian Institute for Health, Brussels, Belgium; 2 Laboratory of Immunology, Department of Translational Physiology, Infectiology and Public Health, Faculty of Veterinary Medicine, Ghent University, Merelbeke, Belgium; 3 Division of Preventive Health Policy, Flemish Department of Care, Environmental Healthcare, Brussels, Belgium; 4 Infectious Disease Surveillance Unit, Agence pour une Vie de Qualité (AVIQ), Charleroi, Belgium; 5 Centre for Infectious Disease Control, National Institute for Public Health and Environment (RIVM), Bilthoven, The Netherlands

## Abstract

**Background:**

Ticks carry a variety of microorganisms, some of which are pathogenic to humans. The human risk of tick-borne diseases depends on, among others, the prevalence of pathogens in ticks biting humans. To follow-up on this prevalence over time, a Belgian study from 2017 was repeated in 2021.

**Methods:**

During the tick season 2021, citizens were invited to have ticks removed from their skin, send them and fill in a short questionnaire on an existing citizen science platform for the notification of tick bites (TekenNet). Ticks were morphologically identified to species and life stage level and screened using multiplex qPCR targeting, among others, *Borrelia burgdorferi* (sensu lato), *Anaplasma phagocytophilum*, *Borrelia miyamotoi*, *Neoehrlichia mikurensis*, *Babesia* spp., *Rickettsia helvetica* and tick-borne encephalitis virus (TBEV). The same methodology as in 2017 was used.

**Results:**

In 2021, the same tick species as in 2017 were identified in similar proportions; of 1094 ticks, 98.7% were *Ixodes ricinus*, 0.8% *Ixodes hexagonus* and 0.5% *Dermacentor reticulatus*. A total of 928 nymphs and adults could be screened for the presence of pathogens. *Borrelia burgdorferi* (s.l.) was detected in 9.9% (95% CI 8.2–12.0%), which is significantly lower than the prevalence of 13.9% (95% CI 12.2–15.7%) in 2017 (*P* = 0.004). The prevalences of *A. phagocytophilum* (4.7%; 95% CI 3.5–6.3%) and *R. helvetica* (13.3%; 95% CI 11.2–15.6%) in 2021 were significantly higher compared to 2017 (1.8%; 95% CI 1.3–2.7% and 6.8%; 95% CI 5.6–8.2% respectively) (*P* < 0.001 for both). For the other pathogens tested, no statistical differences compared to 2017 were found, with prevalences ranging between 1.5 and 2.9% in 2021. *Rickettsia raoultii* was again found in *D. reticulatus* ticks (*n* = 3/5 in 2021). Similar to 2017, no TBEV was detected in the ticks. Co-infections were found in 5.1% of ticks. When combining co-infection occurrence in 2017 and 2021, a positive correlation was observed between *B. burgdorferi* (s.l.) and *N. mikurensis* and *B. burgdorferi* (s.l.) and *B. miyamotoi* (*P* < 0.001 for both).

**Conclusions:**

Although the 2021 prevalences fell within expectations, differences were found compared to 2017. Further research to understand the explanations behind these differences is needed.

**Graphical Abstract:**

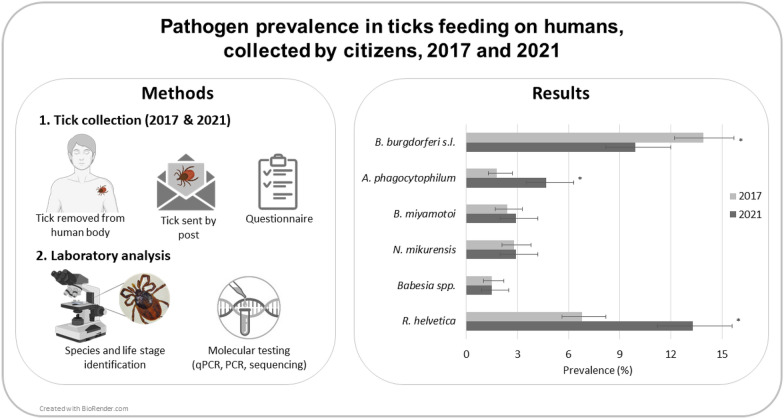

**Supplementary Information:**

The online version contains supplementary material available at 10.1186/s13071-024-06427-x.

## Background

Ticks are important vectors of a variety of microorganisms comprising bacteria, viruses and parasites. In Europe, the most common tick-borne disease (TBD) in humans is Lyme borreliosis, caused by bacteria of the *Borrelia burgdorferi* (sensu lato) complex [1].

In Belgium, the incidence of erythema migrans, the most common manifestation of Lyme borreliosis, in the period 2015–2017 was estimated at 97.6 cases/100,000 inhabitants per year [2]. Annual differences in the epidemiology of the disease, which correlate to variations in climate and exposure of the population to tick bites, are observed, with no overall increasing or decreasing trend over the past decade [3].

Besides *B. burgdorferi* (s.l.), other less prevalent pathogens such as tick-borne encephalitis virus (TBEV), *Anaplasma phagocytophilum*, *Borrelia miyamotoi*, *Neoehrlichia mikurensis*, *Babesia* spp. and *Rickettsia* spp. can cause disease in humans, often as asymptomatic or mild self-limiting infections. Possible symptoms include flu-like illnesses, but more severe complications can occur and some infections can be fatal [4, 5]. In Belgium, rare autochthonous cases of confirmed TBEV and confirmed anaplasmosis are reported, albeit both diseases are probably underdiagnosed [6, 7]. For spotted fever rickettsia such as *Rickettsia raoultii* and *R. monacensis*, no confirmed autochthonous cases have been reported up to now in Belgium [8]. For *B. miyamotoi* disease and neoehrlichiosis, no cases at all have been notified, yet no specific surveillance systems are in place for these pathogens. Although no clinical cases of babesiosis have been reported, antibodies have been found in studies in symptomatic patients [9].

Some other bacteria such as *R. helvetica* and *Spiroplasma ixodetis* are considered emerging tick-borne micro-organisms but the true association with disease remains unclear, with only a few cases described so far [10–12].

Although present in ticks, for some pathogens, such as *Francisella tularensis* and *Coxiella burnetii*, ticks are not the only transmission route, but sporadic cases of an ulcero-glandular form of tularemia in Belgium were notified as linked to a tick bite [13].

Overall, surveillance data on TBD in humans, other than Lyme borreliosis, are still scarce in Belgium, with possible underdiagnosis and under-reporting. To increase knowledge on the risk and exposure of the Belgian population to ticks and TBD, a citizen science platform (www.tekennet.sciensano.be) was set up in 2015 where citizens are invited to report their tick bites. Indeed, citizen science has been shown to be an effective tool for environmental and health research as it allows the collection of data across a wide area and period, is cost-effective and enables obtaining non-traditional data [14]. It has commonly been used for the surveillance of tick bites and to research tick populations and tick-borne pathogens in Europe and the US [15–21]. Likewise, in 2017 the Belgian project TekenNet invited citizens to send ticks removed from their skin to be analyzed for the presence of pathogens as this is an important indicator of the human risk of TBD [22]. The current study aims to repeat the study performed in 2017 to follow up on possible changes over time in the prevalence of these tick-borne pathogens.

## Methods

### Tick collection

The same methodology was used for the collection of ticks as in 2017 [22]. Belgian citizens were invited to send ticks removed from humans to the Belgian Health Institute Sciensano between April 1 and October 31, 2021. Ticks could be sent, collated on a sheet of paper, free of charge by postal mail. In addition, participants were asked to fill in a questionnaire on the citizen science platform TekenNet.be to allow the collection of additional epidemiological information such as the birth year and residence of the bitten person, the date of the bite, the geographical location and environment of the bite site and the type of activity performed when the bite took place. All received envelopes were stored at − 80 °C until analysis. As in 2017, awareness of the study was raised through a press release and the website TekenNet.be. Ticks were excluded from the analysis if the bite did not occur within the defined timeframe, if the tick was removed from an animal (based on information in the questionnaire), if the tick was not attached to the skin (i.e. found before biting or in nature) and if no information on geographical location was available at all.

### Tick identification, pre-analytical sample processing and nucleic acids extraction

Ticks were identified morphologically to the species level and developmental stage using standard taxonomic keys [23, 24]. The individual ticks were then washed in ethanol 70% for 2 min, washed twice in distilled water for an additional 2 min each and then placed in a 1.5 ml microcentrifuge tube containing 450 µl of Dulbecco’s modified Eagle’s medium (DMEM) (41965-039, Gibco^®^, Rockville, MD, USA). Two stainless steel beads (5 mm, 69989, Qiagen^®^, Aarhus, Denmark) were inserted into the tube together with 50 µl of chitinase (5 mg/ml, C6137-25UN, Sigma-aldrich^®^, Burlington, MA, USA). After 30 min of incubation, the tubes were placed in a TissueLyser (85300, Qiagen^®^, Aarhus, Denmark) and homogenized at 20 Hz for 5 min.

Total nucleic acids were extracted from 200 µl of tick homogenate using the MagMax Total Nucleic Acids Isolation Kit (AM1840, Applied Biosystems™, Waltham, MA, USA) following the manufacturer’s protocol. The eluted nucleic acids (50 µl) were preserved at − 20 °C before further processing.

### Tick species confirmation and pathogen detection by PCR

All real-time quantitative PCRs (qPCR) were carried out on a LightCycler^®^ 480 (Roche Diagnostics Nederland B.V, Almere, The Netherlands). The primers and probes designed for *B. burgdorferi* (s.l.), *B. miyamotoi*, *A. phagocytophilum*, *Babesia* spp., *Babesia microti*, *R. helvetica*, *N. mikurensis*, *S. ixodetis*, *C. burnetii*, *F. tularensis*, TBEV and *Ixodes*/*Dermatocentor* genera were those as previously described [22, 25–28]. The list of the entire primer sets, mix components and run cycles is provided in Additional file 1 (Tables S1 and S2). *Ixodes*/*Dermatocentor* qPCR was performed as an internal control for the presence of inhibitors in the reaction mix and confirm the genus of the identified tick. The qPCRs for pathogens were triple multiplexed.

Positive samples for *Borrelia* spp., *Rickettsia* spp. not confirmed to be *R. helvetica* and *Babesia* spp. were selected for sequencing to identify the species. For this purpose, a conventional PCR on a Biometra T Gradient thermocycler (Biometra, Göttingen, Germany) was used. The primers, mix and run cycles used for these PCRs are described in Additional file 1 (Table S2).

The amplicons obtained with conventional PCRs were analyzed on a 2% agarose gel. If visualized, the PCR product was subcontracted for Sanger sequencing at the Genewiz company (Germany).

### Statistical analyses

All analyses were conducted in R 4.2.1 [29]. The proportions of tick species and life stages in 2021 were compared to those in 2017 using Pearson’s Chi-squared tests. For each pathogen, the total prevalence as well as prevalences by life stage were compared between 2017 and 2021 using Pearson’s Chi-squared tests or Fisher’s exact tests when appropriate (frequency < 5). To analyze co-infections and differences in pathogen prevalence by age class of the persons bitten, region, period (months), type of environment and type of activity, data of 2017 and 2021 were combined to increase the sample size and power. In the latter, logistic regressions considering year as a possible confounder were performed. In addition, data from 2021 were analyzed separately, using Pearson’s Chi-squared tests or Fisher’s exact tests when appropriate, as was done in 2017 (Additional file 2: Tables S3 and S4). *P*-values < 0.05 were considered significant.

## Results

### Tick species and life stages in 2021 vs. 2017

**Fig. 1:**
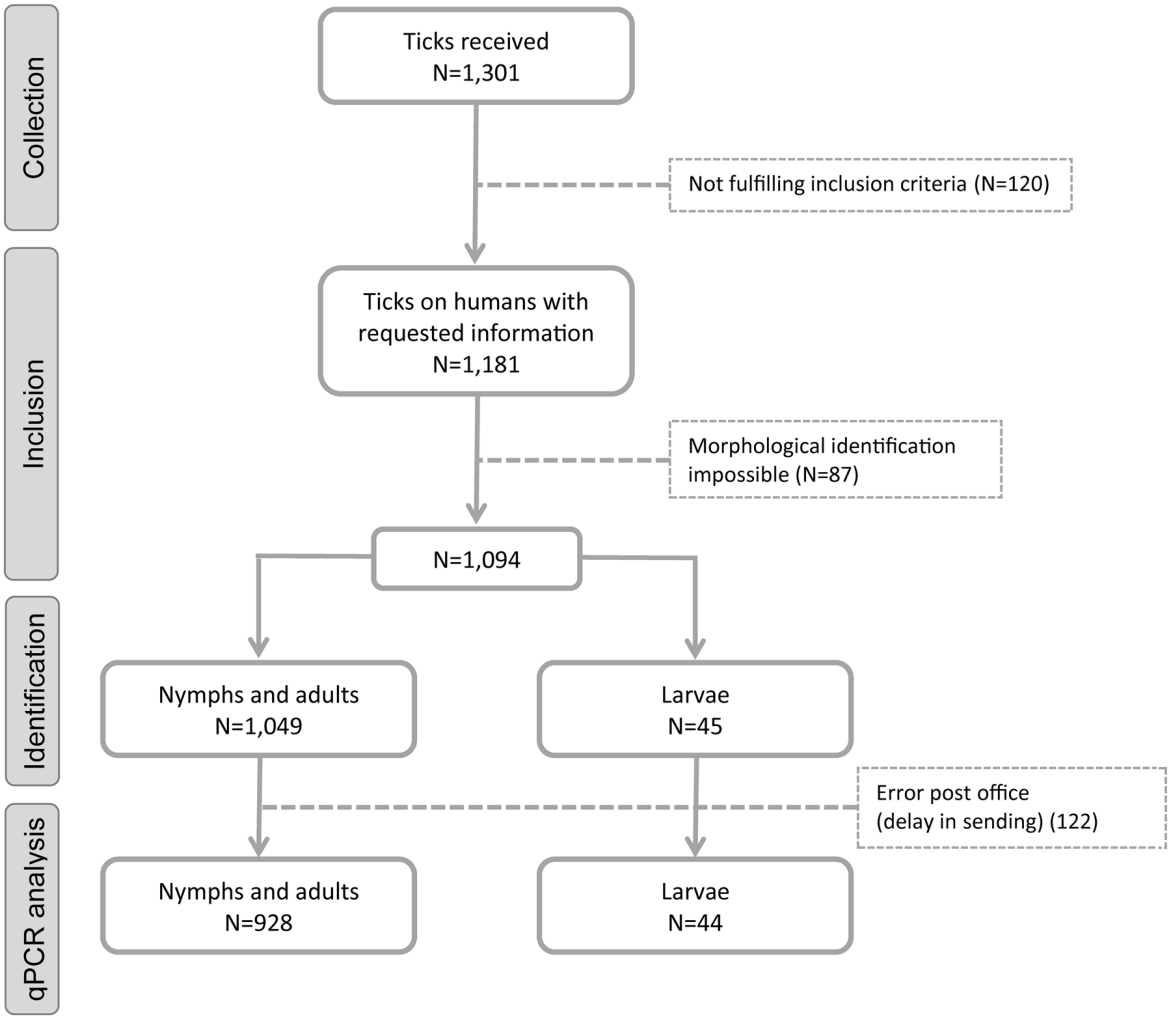
Tick inclusion flowchart

In 2021, a total of 1301 ticks were collected between April 1 and October 31. After the exclusion of ticks that did not fulfill the inclusion criteria (*n* = 120) and unidentifiable ticks (too damaged) (*n* = 87), 1094 ticks collected—presumably—from humans remained for the morphological identification of the species and determination of the life stage (Fig. 1). The large majority of these ticks were *Ixodes ricinus* (98.7%), nine ticks were *I. hexagonus* (0.8%) and five were *Dermacentor reticulatus* (0.5%). All together, they comprised 896 nymphs (81.9%), 139 adult females (12.7%), 14 adult males (1.3%) and 45 larvae (4.1%) (all species). In 2017, when 1599 ticks were included, the same three tick species were identified in similar proportions (*χ*^2^ = 0.728, *df* = 2, *P* = 0.695), yet the distribution of the life stages differed with fewer nymphs in 2017 (76.6% vs. 81.9%) (*χ*^2^ = 13.292, *df* = 3, *P* = 0.004, all life stages compared, Fig. 2a). Over time, the proportion of nymphs on the total number of ticks received in a month (all species) was highest in May in 2021 (87.7%), whereas in 2017 this was in September (81.0%) (Fig. 2b, c). In both years, most ticks were sent in May, June (peak) and July (Fig. 2c). As in 2017, the age of the persons bitten varied widely (range 1–98 years; median 53 in 2021) with an underrepresentation of persons aged 15 to 24 years old (4% in 2021).

**﻿Fig. 2:**
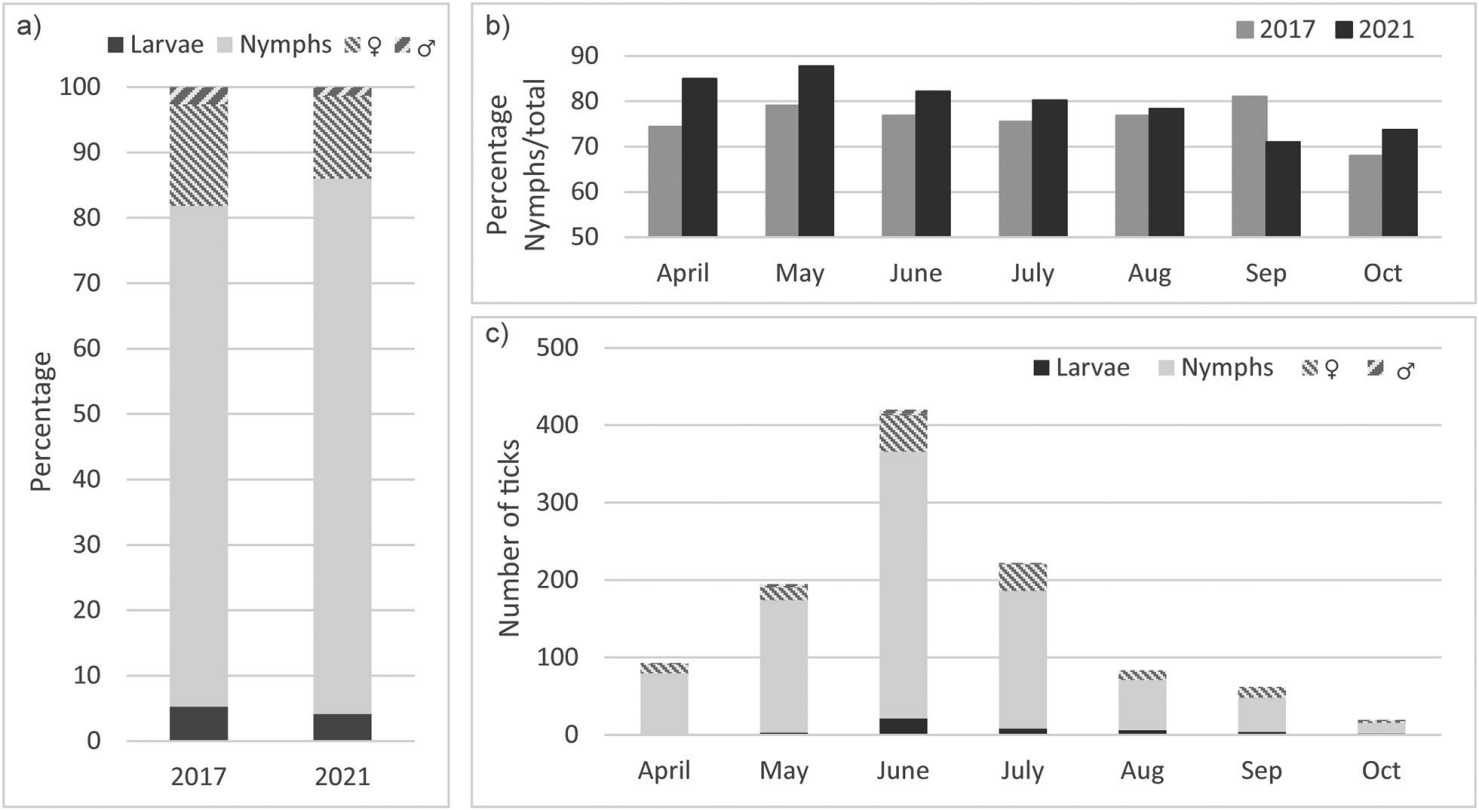
**a** Proportion of ticks identified by life stage in 2017 and 2021 (all species). **b** Proportion of nymphs identified by month in 2017 and 2021 (all species). **c** Number of ticks identified by life stage and month in 2021 (all species)

### Pathogen prevalence

Of the 1049 nymphs and adult ticks identified in 2021, 928 were analyzed for the presence of tick-borne pathogens (792 nymphs, 136 adults) (Fig. 1). Of these, 915 were *I. ricinus* (98.6%) (786 nymphs, 129 adults), eight *I. hexagonus* (six nymphs, two adults) and five *D. reticulatus* (all adults). Analyzable larvae (*n* = 44) were tested and reported separately. The other ticks were excluded from the pathogen analysis as they arrived in the laboratory with a 1- to 5-month delay due to a postal error. Among the ticks that did not fulfill the inclusion criteria, there were seven soft ticks of the species *Argas reflexus* (family *Argasidae*), sent by one person. Even though these ticks were not included in the general pathogen analysis (not removed from the skin and some were suspected of having bitten outside the study period), they were analyzed separately as it was, to our knowledge, the first time that soft ticks were reported to, possibly, have bitten a human in Belgium.

#### *Borrelia burgdorferi* (s.l.) prevalence in 2021 vs. 2017

*Borrelia burgdorferi* (s.l.) was detected in 9.9% (95% CI 8.2–12.0%) of nymphs and adult ticks, which is significantly lower than the prevalence of 13.9% (95% CI 12.2–15.7%) observed in 2017 (*χ*^2^ = 8.278, *df* = 1, *P* = 0.004) (Table 1). As in 2017, in 2021, nymphs were less often infected than adult ticks, with a prevalence of 8.5% and 18.4%, respectively (*χ*^2^ = 11.709, *df* = 1, *P* < 0.001) (Table 1). One of the eight *I. hexagonus* ticks (adult female) and none of the *D. reticulatus* ticks were infected with *B. burgdorferi* (s.l.). Of the 92 qPCR-positive *B. burgdorferi* (s.l.) ticks, genospecies could be determined in 27% (*n* = 25). The most frequently detected genospecies was *B. afzelii* (72.0%, *n* = 18), followed by *B. burgdorferi* s.s. (12.0%, *n* = 3), *B. garinii* (8.0%, *n* = 2) and *B. bavariensis* and *B. valaisiana* (both 4%, *n* = 1). Due to the small number of successful genospecies identifications, statistical comparison with 2017 (52% *B. afzelii*) was not possible. As in 2017, no larvae were infected with *B. burgdorferi* (s.l.). In the separate analysis of *A. reflexus* ticks, none were positive for *B. burgdorferi* (s.l.).

**Table 1:** Pathogen prevalence in feeding ticks on humans in 2017 (1225 nymphs and 290 adults) and 2021 (792 nymphs and 136 adults) by life stage (all species)

Pathogen	Tick life stage	2017	2021	*P*-value 2021 vs. 2017
*Borrelia burgdorferi* (s.l.)	Nymphs	12.3 (10.6–14.3)	8.5 (6.7–10.6)	0.006*
	Adults	20.3 (16.1–25.4)^b^	18.4 (12.7–25.8)^b^	0.635
	Total	13.9 (12.2–15.7)	9.9 (8.2–12.0)	0.004*
*Anaplasma phagocytophilum*	Nymphs	1.6 (1.1–2.5)	4.5 (3.3–6.2)	< 0.001*
	Adults	2.8 (1.4–5.4)	5.9 (3.0–11.3)	0.114
	Total	1.8 (1.3–2.7)	4.7 (3.5–6.3)	< 0.0001*
*Borrelia miyamotoi*	Nymphs	2.6 (1.9–3.7)	2.9 (1.9–4.3)	0.694
	Adults	1.4 (0.5–3.6)	2.9 (1.1–7.6)	0.273
	Total	2.4 (1.7–3.3)	2.9 (2.0–4.2)	0.420
*Neoehrlichia mikurensis*	Nymphs	3.0 (2.2–4.1)	2.7 (1.7–4)	0.628
	Adults	2.1 (0.9–4.5)	4.4 (2.0–9.5)	0.173
	Total	2.8 (2.1–3.8)	2.9 (2.0–4.2)	0.918
*Babesia* spp.	Nymphs	1.4 (0.9–2.2)	1.5 (0.9–2.6)	0.814
	Adults	1.7 (0.7–4.1)	1.5 (0.4–5.7)	1
	Total	1.5 (1.0–2.2)	1.5 (0.9–2.5)	0.910
*Rickettsia helvetica*	Nymphs	6.9 (5.6–8.4)	13.5 (11.3–16.1)	< 0.0001*
	Adults	6.6 (4.2–10.0)	11.8 (7.3–18.3)	0.068
	Total	6.8 (5.6–8.2)	13.3 (11.2–15.6)	< 0.0001*
*TBEV*	Nymphs	0	0	Inf
	Adults	0	0	Inf
	Total	0	0	Inf
*Any pathogen*^a^	Nymphs	24% (21.7–26.5)	28.7% (25.6–31.9)	0.020*
	Adults	30% (25–35.5)^c^	39% (31.1–47.4)^c^	0.066
	Total	25.1 (23.0–27.4)	30.2 (27.3–33.2)	0.007*

^a^Any of the infections above or *Rickettsia raoultii*

^b^Statistically significant different in adults compared to nymphs within the same year (*P* < 0.05)

^c^Statistically significant different in adults compared to nymphs within the same year (*P* < 0.001)

^*^Significant *P*-values

#### Other tick-borne pathogen prevalence in 2021 vs. 2017

The prevalence of *A. phagocytophilum* (4.7%; 95% CI 3.5–6.3%) and *R. helvetica* (13.3%; 95% CI 11.2–15.6%) in nymphs and adult ticks collected in 2021 was significantly higher compared to 2017 (*A. phagocytophilum*: *χ*^2^ = 16.841, *df* = 1, *P* < 0.001; *R. helvetica*: *χ*^2^ = 28.568, *df* = 1, *P* < 0.001) (Table 1). For *Babesia* spp., *B. miyamotoi* and *N. mikurensis*, the 2021 prevalence ranged between 1.5 and 2.9%; no statistically significant differences to 2017 were found (Table 1). No statistical differences in infection rates between nymphs and adults were found for any of these tick-borne pathogens, both in 2017 and 2021 (Table 1). Species could be determined for 57% (*n* = 8) of the *Babesia* spp.positive ticks, consisting of *B. venatorum* (75%, *n* = 6) and *B. capreoli* (25%, *n* = 2). In 2017, *B. divergens* and *B. microti* were also found. Three of five *D. reticulatus* ticks (all adult female) were infected with *R. raoultii*, which is similar to 2017 (2/5 infected *D. reticulatus* ticks). One out of eight *I. hexagonus* nymphs was infected with *R. helvetica* (nymph)*.* Two *I. ricinus* larvae were infected with *Babesia* spp., one with *A. phagocytophilum* and one with *R. helvetica*. In both years, no TBEV was detected in the sample.

*Francisella tularensis*, *C. burnetii* and *S. ixodetis* were also detected in the 2021 tick sample with the highest prevalence for the latter. As their transmission by ticks to humans is either unconfirmed or rare, the results of the analysis on their prevalences are provided in Additional file 3 (Tables S5 and S6).

#### Prevalence by characteristics, 2017 and 2021 combined

Table 2 shows the prevalence of the pathogens according to the characteristics of the person bitten and the circumstances in which the bite took place in the combined data of 2017 and 2021. For *B. burgdorferi* (s.l.), no significant differences were found in the prevalence by any of the researched characteristics (age category, region, period (months), type of environment and type of activity). In the analysis of the prevalence of the other pathogens (*A. phagocytophilum*, *B. miyamotoi*, *N. mikurensis, Babesia* spp. and *R. helvetica*), no significant differences were found by age category of the person bitten or by region where the bite took place (Flanders, Wallonia or Brussels).Yet, significant differences were found by period (months), with a higher prevalence of *Babesia* spp. observed in ticks in September–October compared to April–June (*P* = 0.018) or July–August (*P* = 0.003) (Table 2) and a higher prevalence of *A. phagocytophilum* in April–June compared to July–August (*P* = 0.038) (Table 2). By type of environment, differences were found in the prevalence of *Babesia* spp., namely a higher prevalence in ‘grassland, agricultural field’ compared to ‘garden’ (*P* = 0.006) (Table 2). The separate analysis of the 2021 data is added in Additional file 2 (Table S3).

**Table 2:** Pathogen prevalence in feeding ticks on humans in 2017 and 2021 combined, according to demographic and other characteristics

	*Borrelia burgdorferi* (s.l.) % pos (95% CI)	*Anaplasma phagocytophilum* % pos (95% CI)	*Borrelia miyamotoi* % pos (95% CI)	*Neoehrlichia mikurensis* % pos (95% CI)	*Babesia* spp. % pos (95% CI)	*Rickettsia helvetica* % pos (95% CI)
Age	*P* = 0.244	*P* = 0.806	*P* = 0.120	*P* = 0.328	*P* = 0.471	*P* = 0.575
< 15 (*n* = 471)	13.6 (10.8–17)	2.1 (1.1–3.9)	3.0 (1.8–5)	3.2 (1.9–5.2)	0.6 (0.2–2)	8.3 (6.1–11.1)
15–24 (*n* = 112)	6.2 (3–12.5)	1.8 (0.4–6.9)	0 (0–3.2)	1.8 (0.4–6.9)	1.8 (0.4–6.9)	10.7 (6.2–17.9)
25–44 (*n* = 517)	12.8 (10.2–15.9)	2.9 (1.8–4.8)	2.3 (1.3–4)	3.5 (2.2–5.5)	1.7 (0.9–3.3)	10.3 (7.9–13.2)
45–64 (*n* = 755)	12.7 (10.5–15.3)	3.2 (2.1–4.7)	2.3 (1.4–3.6)	3.3 (2.2–4.9)	1.7 (1–2.9)	7.8 (6.1–10)
65+ (*n* = 552)	12.1 (9.7–15.1)	3.6 (2.3–5.5)	3.3 (2.1–5.1)	1.8 (1–3.3)	1.6 (0.9–3.1)	10.7 (8.4–13.6)
Region	*P* = 0.231	*P* = 0.397	*P* = 0.835	*P* = 0.384	*P* = 0.574	*P* = 0.113
Brussels (*n* = 28)	3.6 (0.5–21.4)	0 (0–12.3)	3.6 (0.5–21.4)	0 (0–12.3)	0 (0–12.3)	7.1 (1.8–24.5)
Flanders (*n* = 1358)	12.7 (11.1–14.6)	3.0 (2.2–4.1)	2.7 (1.9–3.7)	3.1 (2.3–4.2)	1.4 (0.9–2.2)	10.1 (8.6–11.8)
Wallonia (*n* = 1020)	12.4 (10.5–14.5)	2.7 (1.9–3.9)	2.4 (1.6–3.5)	2.7 (1.9–3.9)	1.7 (1–2.7)	7.9 (6.4–9.8)
Period (months)	*P* = 0.449	*P* = 0.024*	*P* = 0.402	*P* = 0.696	*P* = 0.017*	*P* = 0.955
April–June (*n* = 1534)	12.8 (11.3–14.6)	3.7 (2.8–4.7)^a^	2.7 (2–3.7)	3.1 (2.3–4.1)	1.5 (1–2.2)^b^	9.3 (8–10.9)
July–August (*n* = 739)	11.2 (9.1–13.7)	1.9 (1.1–3.2)^a^ (p = 0.038)	2.6 (1.6–4)	2.4 (1.5–3.8)	0.8 (0.4–1.8)^b^	9.2 (7.3–11.5)
September–October (*n* = 170)	12.9 (8.7–18.9)	1.2 (0.3–4.6)	1.2 (0.3–4.6)	2.9 (1.2–6.9)	4.1 (2–8.4)^b^	8.8 (5.4–14.1)
Type of environment	*P* = 0.801	*P* = 0.440	*P* = 0.147	*P* = 0.211	*P* = 0.037	*P* = 0.342
Wood/forest (*n* = 776)	12.4 (10.2–14.9)	2.8 (1.9–4.3)	2.3 (1.5–3.7)	3.6 (2.5–5.2)	2.1 (1.3–3.3)	9.1 (7.3–11.4)
Garden (*n* = 1075)	12.5 (10.6–14.6)	2.8 (2–4)	3.2 (2.3–4.4)	2.1 (1.4–3.2)	1.1 (0.6–2)^c^	8.5 (6.9–10.3)
Nature reserve, not forest (*n* = 173)	15.6 (10.9–21.8)	5.8 (3.1–10.4)	1.7 (0.6–5.2)	4.6 (2.3–9)	1.2 (0.3–4.5)	12.1 (8–17.9)
Grassland, agricultural field (*n* = 104)	10.6 (6–18.1)	2.9 (0.9–8.6)	0 (0–3.5)	1.9 (0.5–7.4)	4.8 (2–11)^c^	14.4 (8.9–22.6)
Other (*n* = 73)	12.3 (6.5–22)	1.4 (0.2–9.1)	1.4 (0.2–9.1)	1.4 (0.2–9.1)	0 (0–4.9)	12.3 (6.5–22)
Unknown (*n* = 206)	11.2 (7.5–16.2)	2.4 (1–5.7)	2.4 (1–5.7)	3.9 (2–7.6)	0.5 (0.1–3.4)^c^	7.3 (4.4–11.7)
Activity of person bitten	*P* = 0.439	*P* = 0.165	*P* = 0.867	*P* = 0.484	*P* = 0.262	*P* = 0.038*
Leisure (*n* = 2052)	12.9 (11.5–14.4)	2.9 (2.3–3.7)	2.5 (1.9–3.3)	2.7 (2.1–3.5)	1.5 (1.1–2.1)	9.5 (8.3–10.8)^d^
Professional (*n* = 84)	10.7 (5.7–19.3)	7.1 (3.2–15)	3.6 (1.2–10.5)	3.6 (1.2–10.5)	1.2 (0.2–8)	10.7 (5.7–19.3)^d^
Other (*n* = 133)	10.5 (6.3–17)	2.3 (0.7–6.8)	3.0 (1.1–7.7)	5.3 (2.5–10.6)	0 (0–2.7)	4.5 (2–9.7)^d^
Unknown (*n* = 118)	8.5 (4.6–15)	1.7 (0.4–6.5)	1.7 (0.4–6.5)	3.4 (1.3–8.7)	1.7 (0.4–6.5)	6.8 (3.4–13)

^a^Significantly higher in April–June compared to July–August (*P* = 0.038)

^b^Significantly higher in September–October compared to April–June (*P* = 0.018) and compared to July–August (*P* = 0.003)

^c^Significantly higher in ‘grassland, agricultural field’ compared to ‘garden’ (*P* = 0.006) and compared to ‘unknown’ (*P* = 0.035)

^d^Significantly higher in category ‘leisure’ (*P* = 0.017) and ‘professional’ (*P* = 0.042) compared to ‘other’

*Significant *P*-values

#### Co-infections

In 2021, 5.1% of nymphs and adult ticks (47/928) were infected with multiple pathogens compared to 3.9% (59/1515) in 2017 (*P* = 0.168). All were *I. ricinus*. More precisely, 44 ticks were positive for two pathogens (4.8%) and three ticks carried three pathogens (0.3%) (Table 3).

**Table 3:** Number of ticks (co-)infected by year

Number of pathogens	2017	2021
0	1134 (74.8%)	648 (69.9%)
1	322 (21.3%)	233 (25.1%)
2	55 (3.6%)	44 (4.7%)
3	4 (0.3%)	3 (0.3%)

In the combined data of 2017 and 2021, the two most common co-infections were *B. burgdorferi* (s.l.) + *N. mikurensis* (*n* = 27) and *B. burgdorferi* (s.l.) + *R. helvetica* (*n* = 26) (Table 4). Ticks infected with either *N. mikurensis* or *Babesia* spp. were most often co-infected with other pathogens (45.7% and 41.8% respectively) (Table 4). Infections with *N. mikurensis* and *B. miyamotoi* were observed significantly more frequently in *B. burgdorferi* (s.l.) positive ticks than in *B. burgdorferi* (s.l.) negative ticks (8.9% vs. 2.0% for *N. mikurensis*, *P* < 0.001 and 5.6% vs. 2.1% for *B. miyamotoi*, *P* < 0.001) (Table 4). In addition, a positive correlation was observed between *Babesia* spp. and *R. helvetica* infections (*P* = 0.04) (Table 4).

**Table 4:** Number of ticks co-infected by pathogen in 2017 and 2021 combined

	*N* co-infected/*N* infected (%)	*Borrelia burgdorferi* (s.l.)	*Anaplasma phagocytophilum*	*Borrelia miyamotoi*	*Neoehrlichia mikurensis*	*Babesia* spp.	*Rickettsia helvetica*
*B. burgdorferi* (s.l.)	81^a^/302 (26.8%)	–	12	17***	27***	6	26
*A. phagocytophilum*	23/72 (31.9%)	12	–	1	1	0	12
*B. miyamotoi*	20/63 (31.7%)	17***	1	–	0	1	5
*N. mikurensis*	32/70 (45.7%)	27***	1	0	–	2	3
*Babesia* spp.	15^b^/36 (41.7%)	6	0	1	2	–	7*
*R. helvetica*	48/226 (21.2%)	26	12	5	3	7*	–

*P < 0.05; **P < 0.01; ***P < 0.001

^a^*Borrelia afzelii* (*n* = 30), *B. garinii* (*n* = 3), *B. spielmanii* (*n* = 3), *B. burgdorferi* s.s. (*n* = 1), unknown genospecies (*n* = 44). Co-infections of different genospecies of *B. burgdorferi* (s.l.) could not be identified as the qPCR did not target each individual genospecies

^b^*Babesia venatorum* (*n* = 10), *B. microti* (*n* = 2), *B. capreoli* (*n* = 1), unknown genospecies (*n* = 2). Co-infections of different genospecies of *Babesia* spp. could not be identified as the qPCR did not target each individual genospecies

In the analysis of the 2021 data separately (Additional file 2: Table S4), in addition, a positive correlation was observed between *B. burgdorferi* (s.l.) and *Babesia* spp. (*P* = 0.04), which was not observed in the combined data or in 2017.

## Discussion

Several European studies have investigated the presence of tick-borne pathogens in ticks collected from humans, but the large majority concern ticks collected from patients consulting a physician or ticks sent for diagnostic purposes [30–36]. By collecting ticks from the general population across the whole country, the results of the current study provide a good indication of the risk of acquiring TBD after a tick bite in Belgium. As the current study repeats an earlier study performed in 2017, it allows comparing results between both years [22].

As expected, no differences were found between the two years in terms of tick species, with most collected ticks identified as *I. ricinus* (99%) and only a few *I. hexagonus* and *D. reticulatus*. Of all ticks in 2021, 82% were nymphs, which is significantly more than in 2017 (77%). These proportions are similar to a study on *I. ricinus* ticks removed from humans from one county in Romania (average of 80% nymphs) but higher than in some other studies on ticks from humans (69.9% nymphs in Slovakia, 68.7% in Poland, 66.8% in Germany, 59.8% in northwest Italy and 53.2% in The Netherlands) [32, 33, 35, 37, 38]. On the other hand, large fluctuations in the proportion of nymphs between (sometimes consecutive) years were also described in some of these studies [35, 37]. Note that such differences in life stages between studies can influence the pathogen prevalence and complicate comparison; also in our study, it was shown that the infection rate in adult ticks was significantly higher than in nymphs (Table 1). The latter is not unexpected as adults have fed on several hosts during their life cycle, which leads to an increased infection rate compared to nymphs [33, 35].

In 2021, 9.9% of the analyzed nymphs and adult ticks were infected by *B. burgdorferi* (s.l.), which is significantly lower than in 2017 (13.9% infected). Both results fall within the ranges of *B. burgdorferi* (s.l.) prevalence described in previous literature on ticks collected from humans in Europe, going from 6.4% in a study in N–W Italy to 26% in a study from Germany and even 29% in a study in The Netherlands, yet the latter included some erythema migrans patients [32, 34, 36–40]. In questing ticks in Belgium, widely varying prevalences ranging from 2.8 to 37% have been reported previously [41–44]. Yet, these ticks were often collected at specific geographic locations or during a short time period only, which can have an important impact on the prevalence estimates. As in most European countries, *B. afzelii* was the most frequently detected *Borrelia* genospecies in 2021 [35, 45, 46].

Only few studies have researched the evolution of *B. burgdorferi* (s.l.) prevalence over time. On ticks removed from humans, annual differences but no trends have been reported by a German study performed between 2013 and 2017 with 17.2% of ticks carrying *Borrelia* spp. (including *B. miyamotoi*) in 2014, compared to 24.1% in 2015 [35]. Other research mainly focused on questing ticks. A study from The Netherlands reported a slight significant decrease in the *B. burgdorferi* (s.l.) prevalence in *I. ricinus* nymphs between 2009 and 2016 [45], but in the city of Hanover in Germany, where questing ticks have been collected for 15 years, the overall *B. burgdorferi* (s.l.) prevalence remained stable. However, this might be due to the more local tick collection [47]. Whether the lower *B. burgdorferi* (s.l.) prevalence in 2021 in our study reflects a trend over time needs further research. Yearly fluctuations in the infection prevalence of pathogens in ticks have been shown previously [35, 40]. These could be caused by yearly differences in a wide range of ecological and climatological factors, influencing the tick vector, reservoir hosts and pathogen presence [48]. It is important to consider that such differences also exist between countries and even at specific locations within countries, complicating comparison of pathogen prevalences in ticks. In addition, infection prevalences and trends in questing ticks do not necessarily correspond to those in ticks biting humans, but studies on the latter are less common.

For *A. phagocytophilum* and *R. helvetica*, the current study observed a significantly higher prevalence in 2021 compared to 2017 (increase from 1.8 to 4.7% and 6.8% to 13.3%, respectively). Compared to the other European studies on ticks collected from humans mentioned above, the 2021 *A. phagocytophilum* prevalence was higher than in the studies from The Netherlands (1.0%) and Italy (1.2%) but lower than the one in Romania (5.6%) and Slovakia (13.5%) [31, 32, 40, 49]. Lower prevalences have also been reported in questing ticks in Belgium (0.5–3%) [44, 50, 51], while higher prevalences were reported in ticks removed from animals (5–19.5%) [50, 52]. For *R. helvetica*, compared to our study, the study from Romania observed a lower prevalence of 4.8% while the study from The Netherlands observed a higher prevalence of 19% in ticks from humans. In questing ticks in Belgium, one study focusing on two provinces (6 locations) observed about 7% of ticks positive for *R. helvetica*, while another study on ticks from an area with the highest *Rickettsia* seroprevalence in cattle reported a prevalence of 16.9% [44, 53]. Also across Europe the prevalence in questing ticks varies widely (0–31%) [54–56]. Although *R. helvetica* was the most common pathogen found in the current study, public health relevance remains unclear. Only few human cases have been described in Europe, even though the organism is widespread in ticks [12]. *Rickettsia raoultii*, the causative agent of tick-borne lymphadenopathy, was identified in *D. reticulatus* ticks both in 2017 and 2021 as well as in other European countries [57], but no confirmed autochthonous cases have ever been reported in Belgium [8].

No statistically significant differences in prevalence were found for *Babesia* spp., *B. miyamotoi and N. mikurensis*, between 2017 and 2021. Similar to our result of 1.5% ticks infected by *Babesia* spp., 1.3% of ticks removed from humans in Poland were infected; yet, higher prevalences were observed in the study from Romania (2.9%), The Netherlands (3.5%) and Slovakia (5.2%) [31, 33, 37, 40]. In questing ticks, a study by Azagi et al. [58] in Belgium and The Netherlands found a comparable prevalence of 1.9%. *Babesia venatorum* was, as in 2017, the most prevalent species identified. In contrast to 2017, *B. divergens* and *B. microti* were not found in 2021; for *B. divergens* this could be because only 8 of the 14 *Babesia* spp. positive ticks could be genotyped in 2021; for *B. microti* also in 2017 few ticks were positive (*n* = 2).

The prevalence of *B. miyamotoi* observed in the current study (2.9% in 2021) is similar to the prevalence in the study on ticks removed from humans in The Netherlands (2.3%) and slightly higher than in the Romanian study (1.5%) [31, 37]. It is also slightly higher than the prevalences in questing ticks previously reported in Belgium (1.1–1.6%) [53, 59].

For *N. mikurensis*, the prevalence (also 2.9% in 2021) is slightly lower than what has been found in the other studies on ticks removed from humans mentioned earlier, with 5.4% in The Netherlands, 5.9% in Romania and 4.4% in Slovakia [37, 40]. In questing ticks on specific locations in Belgium, low prevalences of 0.4–1.6% have been reported [53, 60].

TBEV was the only pathogen not detected in the analyzed ticks, both in the current study and in 2017. It has also not been detected in any previous study in ticks in Belgium [61]. However, this does not mean that the virus is not present; rare autochthonous infections do occur, and seroprevalence studies in different animal species reported prevalence rates up to 9.3% (in wild boars), indicating exposure to the virus [61].

Altogether, in 2021, 30.2% (95% CI 27.3–33.2%) of the analyzed nymphs and adult ticks were infected with a pathogen [*Borrelia burgdorferi* (s.l.), *A. phagocytophilum*, *B. miyamotoi*, *N. mikurensis*, *Babesia* spp., *R. helvetica* or *R*. *raoultii*], which is a substantial part. It is also significantly higher than in 2017 (25.1%; 95% CI 23.0–27.4%) but that is in part due to the increase in *R. helvetica*.

Co-infections were found in 5.1% of ticks collected in 2021 and 3.9% in 2017. Direct comparison with other European countries is difficult as the number of searched pathogens varies among studies. Although occurring, co-infections of different genospecies of *B. burgdorferi* (s.l.) could not be identified in our study as the qPCR did not target each individual genospecies, causing the estimated prevalence of co-infections to be an underestimate of the true proportion. The most common co-infections found were *B. burgdorferi* (s.l.) with *N. mikurensis* and *B. burgdorferi* (s.l.) with *R. helvetica*; the latter can be expected because of the high prevalences of both pathogens. The study showed that *N. mikurensis* and *B. miyamotoi* occurred more often in *B. burgdorferi* (s.l.) positive ticks compared to *B. burgdorferi* (s.l.) negative ticks (*P* < 0.001 for both associations, data for 2017 and 2021 combined). A significant association, however less strong, was also found between *Babesia* spp. and *R. helvetica* (*P* = 0.04). Only few studies have statistically analyzed associations between different pathogens in ticks but the positive association between *B. burgdorferi* (s.l.) and *N. mikurensis* has been described previously in studies from, among others, Belgium and The Netherlands, Finland and Norway [52, 62, 63]. To our knowledge, associations between *B. burgdorferi* (s.l.) and *B. miyamotoi* and between *Babesia* spp. and *R. helvetica* have not yet been reported. A positive correlation between *B. burgdorferi* (s.l.) and *Babesia* spp., as observed in our study in 2021 but not in 2017 or the combined data, has previously been described elsewhere, among others in a review from the US by Wasser et al. and a study in Poland on ticks collected from humans [33, 64]. Positive correlations suggest a life cycle involving a common reservoir but could also indicate a possible transmission/proliferation facilitation or a possible survival advantage for both pathogens due to interactions between them, when feeding on a host infected with multiple pathogens, which needs further investigation [52, 64, 65]. In addition, co-infections can result from trans-stadial and, for some pathogens, trans-ovarial transmission, from co-feeding on the same host or from (interrupted) feeding on multiple hosts in one life stage [52]. Co-infections are important to consider as they cause a risk of transmission of multiple pathogens to humans, possibly complicating diagnosis and disease [64].

No statistically significant associations were observed between any of the pathogen prevalences and the age of the person bitten or the region in Belgium. Some seasonal differences were found in the combined data for 2017 and 2021, with a higher prevalence of *Babesia* spp. in the period September–October and of *A. phagocytophilum* in April–June. Other studies on the seasonality of pathogen prevalences in ticks are very scarce but a similar trend for *Babesia* spp. has been reported by a study from Luxembourg and for *A. phagocytophilum* by a study in Norway [66, 67]. The higher prevalence of *Babesia* spp. in ticks for which the bite occurred in ‘grassland, agricultural field’ compared to ‘garden’ could be related to differences in the habitat where the reservoir hosts for *Babesia* spp., reported to be mostly bigger mammals like roe deer and cattle, live [68].

For the first time, soft ticks (*A. reflexus*) were sent by citizens in this study, yet these ticks have been reported previously on pigeons in the same area in Belgium [69]. Although excluded from the general analysis as they were not removed from the skin, they were analyzed and were all negative for any pathogen.

Some limitations need to be considered when interpreting the results of this study. First, the pre-analytical process (i.e. tick storage by citizens/post) was out of our control and could in particular have impacted TBEV detection and success in genospecies sequencing of the tested pathogens. Several ticks had to be excluded as there was an obvious delay of several months in sending the ticks by the post (a problem that was not encountered in 2017); yet, in general, time between tick bite and sending the tick was not controlled for. Second, even though the same qPCRs were used in 2017 and 2021, the analyses were performed in different laboratories that might have their specific laboratory conditions. Third, although no significant differences in pathogen prevalences between regions were found, geographical differences can be present more locally, as seen in other studies [45, 56, 59]. As such, the differences found between 2017 and 2021, e.g. the lower prevalence of *B. burgdorferi* (s.l.) in 2021, are not necessarily present in all provinces or municipalities. Although the tick sampling in 2017 and 2021 is not identical at local geographical level (because of the uncontrolled crowdsourcing of samples), impact on the overall prevalences presented here is expected to be minimal because of the countrywide sampling of many different locations causing a dilution of local effects.

On the other hand, the study has several strengths. As more than seven pathogens were targeted by qPCR in this study, it provides a broad analysis of tick-borne pathogens in ticks removed from humans in Belgium and as such provides a good proxy of the risk for human exposure to pathogens after a tick bite. Nevertheless, the risk on acquiring TBD also depends on other factors such as the number of tick bites, which depends on the abundance of ticks and the exposure of the population to these ticks. An increase in the number of questing ticks can cause an increase in tick bites and can in that way cancel out the effect of a decrease in the infection prevalence in ticks, as reported in a Dutch study [45]. Up to now, in Belgium, annual differences but no increasing or decreasing trends have been observed in the reported number of tick bites (website = tekennet.sciensano.be). By involving citizens in the collection of ticks biting humans, a large number of ticks can be collected in a cost-effective way over several months across a large territory. At the same time, it raises awareness about ticks and TBD among the citizens and promotes their engagement in and trust towards science and scientific research [70].

## Conclusions

In 2021, the same pathogens as in 2017 were tested in ticks removed from humans. Almost one third of ticks were infected with at least one pathogen; yet, this includes a high prevalence of *R. helvetica* for which public health relevance is expected to be limited. Even though the prevalence was lower in 2021 compared to 2017, the most important pathogen for public health remains *B. burgdorferi* (s.l.), with almost 10% of ticks infected in 2021. Whether the observed differences in the prevalence of some pathogens between 2017 and 2021 concern trends over time needs further research. Citizen science has again shown to be an efficient method for the collection of ticks biting humans over the whole of Belgium, allowing evaluation of the risk of exposure to tick-borne pathogens. The current study will be repeated in the future.

## Supplementary Information

Additional file 1. Technical complement information to the multiplex qPCR method. Table S1. Primers and probes by pathogen. Table S2. Summary of mix components and run cycles.

Additional file 2. Analysis of 2021 prevalences by characteristics and co-infections. Table S3. Pathogen prevalence in feeding ticks on humans in 2021, according to demographics and other characteristics. Table S4. Number of ticks co-infected by pathogen in 2021.

Additional file 3. Results for *Spiroplasma ixodetis*, *Francisella tularensis* and *Coxiella burnetii*. Table S5. Pathogen prevalence in feeding ticks on humans in 2017 (1225 nymphs and 290 adults) and 2021 (792 nymphs and 136 adults) by life stage. Table S6. Prevalence of *Spiroplasma ixodetis*, *Francisella tularensis* and *Coxiella burnetii* in feeding ticks on humans in 2021, according to demographics and other characteristics.

## Data Availability

Data supporting the conclusions of this article are included within the article and its additional files. The dataset on the analyzed ticks is available from the corresponding author upon reasonable request. Sanger sequencing sequences were submitted to GenBank. Accession numbers for these sequences are PP874233-PP874257 (*Borrelia burgdorferi* (s.l.)), PP868158-PP868165 (*Babesia* spp.), and PP904449–PP904451 (*Rickettsia raoultii*).

## Abbreviations

DMEM: Dulbecco’s modified Eagle’s medium
s.l.: Sensu lato
s.s.: Sensu stricto
TBD: Tick-borne diseases
TBEV: Tick-borne encephalitis virus
