# Supplementary material for: The prevalence of pathogens in ticks collected from humans in Belgium, 2021, versus 2017
Source: Parasit Vectors. 2024 Sep 5;17:380. doi: 10.1186/s13071-024-06427-x (PMC11378490; doi:10.1186/s13071-024-06427-x)
Supplement: Supplementary file 1 — Additional file 1. Technical complement information to the multiplex qPCR method. Table S1. Primers and probes by pathogen. Table S2. Summary of mix components and run cycles. [file 13071_2024_6427_MOESM1_ESM.docx]

**Additional files to “The prevalence of pathogens in ticks collected from humans in Belgium, 2021 versus 2017”**

**Additional file 1: Technical complement information to the multiplex qPCR method**

**Table S1.** Primers and probes by pathogen.

| Target | Primer FW | Primer REV | Probe | Amplicon length | References |
| --- | --- | --- | --- | --- | --- |
| *A. phagocytophilum* (*ApMSP2*) | ATGGAAGGTAGTGTTGGTTATGGTATT | TTGGTCTTGAAGCGCTCGTA | (Atto425) TGGTGCCAGGGTTGAGCTTGAGATTG (BHQ-1) | 77bp | (1) |
| *B. microti* (*ITS*) | CTCACACAACGATGAAGGACGCA | AACAGAGGCAGTGTGTACAATACA TTCAGA | (Atto520) GCA{G}AATTTAG{C}AAAT{C}AACAGG (BHQ-1) | 103bp | (2) |
| *B. miyamotoi* (*flaB*) | AGAAGGTGCTCAAGCAG | TCGATCTTTGAAAGTGACATAT | (Atto647) AGCACAACAGGAGGGAGTTCAAGC (BHQ-2) | 155bp | (3) |
| *B. burgdorferi s.l* (*ospA*) | AATATTTATTGGGAATAGGTCTAA | CTTTGTCTTTTTCTTTRCTTACA | (Atto520) AAGCAAAATGTTAGCAGCCTTGA (BHQ-1) | 139bp | (4) |
| *B. burgdorferi s.l* (*flaB*) | CAGAIAGAGGTTCTATACAIATTGAIA TAGA | GTGCATTTGGTTAIATTG**Y**GC | (Atto425) CAACTIACAGAIGAAAXTAAIAGAATTGCTGAI CA (BHQ-1) | 89bp | (4) |
| *N. mikurensis* (*groEL*) | CCTTGAAAATATAGCAAGATCAGGTAG | CCACCACGTAACTTATTTAGCACT AAAG | (Atto520) CCTCTACTAATTATTGCXGAAGATGTAGAAGG TGAAGC (BHQ-1) | 99bp | (5) |
| *R. helvetica* (*gltA*) | ATGATCCGTTTAGGTTAATAGGCTTCG  GTC | TTGTAAGAGCGGATTGTTTTCTAG CTGTC | (Atto647) CGATC{C}{A}CG{T}G{C}CGCAGT (BHQ-2) | 89bp | (6) |
| *Rickettsia spp.* (*gltA*) | TCGCAAATGTTCACGGTACTTT | TCGTGCATTTCTTTCCATTGTG | (Atto520) TGCAATAGCAAGAAGAACCGTAGGCTGGATG (BHQ-1) | 74bp | (7) |
| *S. ixodetis* (*rpoB*) | TGTTGGACCAAACGAAGTTG | CCAACAATTGGTGTTTGGGG | (Atto425) GCTAACCGTGCTTTAATGGG (BHQ-1) | 170bp | (8) |
| *Babesia spp.* (*18S*) | CAGCTTGACGGTAGGGTATTGG | TCGAACCCTAATTCCCCGTTA | (Atto520) CGAGGCAGCAACGGMGB | 62bp | (2) |
| *F. tularenisis* (*tul4*) | GCTCCAGAAGGTTCTAAGTG | GCCCAAGTTTTATCGTTCTTC | (Atto 647) TTCTAA GTGCCATGATACAAGCTTCCCAAT TACTAAG (BHQ-2) | 91bp | (9) |
| *C. burnetii* (*IS1111*) | GTCTTAAGGTGGGCTGCGTG | ACTGATCAACTGCGTTGGGA | (Atto425) AGCGAACCATTGGTATCGGACGTTTATG (BHQ-1) | 98bp | (10) |
| TBEV (3'-UTR) | GGG CGG TTC TTG TTC TCC | ACA CAT CAC CTC CTT GTC AGA CT | (FAM) TGAGCCACCATCACCCAGACACA (BHQ1) | 68bp | (11) |
| *Ixodes spp.* (*5S*) | GTCGTAGCCTTCCGTCAGTC | ACGGCATTCCCCTACTGGAT | / | 74bp | (12) |
| *Dermacentor spp.* (*ITS2*) | CGGACACCTGCAGGGAAAG | TTTTGCTAGAGCTCGACGTAC | / | 201bp | (12) |
| *Rickettsia spp* (sequencing) (*gltA*) | GGGGGCCTGCTCACGGCGG | ATTGCAAAAAGTACAGTGAACA | / | 380bp | (13) |
| *Borrelia spp.* (sequencing) (*IGS*) | GAGTTCGCGGGAGAGTAGGTTATTGCC | TCAGGGTACTTAGATGGTTCACTTCC | / | ±390-420 bp | (14) |
| *Babesia spp.* (sequencing) (*18S*) | GTCTTGTAATTGGAATGATGG | TAGTTTATGGTTAGGACTACG | / | 420bp | (15) |

{} = LNA, Y=C or T, I=Inosine, X=position of the quencher on T when not on the first nucleotide and 5’ phosphorylation

**References table S1**

1. Courtney JW, Kostelnik LM, Zeidner NS, Massung RF. Multiplex real-time PCR for detection of *Anaplasma phagocytophilum* and *Borrelia burgdorferi.* J Clin Microbiol. 2004 Jul;42(7):3164–8.

2. Øines Ø, Radzijevskaja J, Paulauskas A, Rosef O. Prevalence and diversity of *Babesia* spp. in questing *Ixodes ricinus* ticks from Norway. Parasit Vectors. 2012 Aug 4;5:156.

3. Zakham F, Jääskeläinen AJ, Castrén J, Sormunen JJ, Uusitalo R, Smura T, et al. Molecular detection and phylogenetic analysis of *Borrelia miyamotoi* strains from ticks collected in the capital region of Finland. Ticks Tick-Borne Dis. 2021 Mar 1;12(2):101608.

4. Heylen D, Tijsse E, Fonville M, Matthysen E, Sprong H. Transmission dynamics of *Borrelia burgdorferi* s.l. in a bird tick community. Environ Microbiol. 2013 Feb;15(2):663–73.

5. Jahfari S, Fonville M, Hengeveld P, Reusken C, Scholte EJ, Takken W, et al. Prevalence of *Neoehrlichia mikurensis* in ticks and rodents from North-west Europe. Parasit Vectors. 2012 Apr 19;5(1):74.

6. de Bruin A, van Leeuwen AD, Jahfari S, Takken W, Földvári M, Dremmel L, et al. Vertical transmission of *Bartonella schoenbuchensis* in Lipoptena cervi. Parasit Vectors. 2015 Mar 21;8(1):176.

7. Boretti FS, Perreten A, Meli ML, Cattori V, Willi B, Wengi N, et al. Molecular Investigations of *Rickettsia helvetica* Infection in Dogs, Foxes, Humans, and *Ixodes* Ticks. Appl Environ Microbiol. 2009 May 15;75(10):3230–7.

8. Krawczyk AI, van Duijvendijk GLA, Swart A, Heylen D, Jaarsma RI, Jacobs FHH, et al. Effect of rodent density on tick and tick-borne pathogen populations: consequences for infectious disease risk. Parasit Vectors. 2020 Jan 20;13(1):34.

9. Versage JL, Severin DDM, Chu MC, Petersen JM. Development of a multitarget Real-Time TaqMan PCR assay for enhanced detection of *Francisella tularensis* in complex specimens. J Clin Microbiol. 2003 Dec;41(12):5492–9.

10. Klee SR, Tyczka J, Ellerbrok H, Franz T, Linke S, Baljer G, et al. Highly sensitive real-time PCR for specific detection and quantification of *Coxiella burnetii*. BMC Microbiol. 2006 Jan 19;6:2.

11. Schwaiger M, Cassinotti P. Development of a quantitative real-time RT-PCR assay with internal control for the laboratory detection of tick borne encephalitis virus (TBEV) RNA. J Clin Virol Off Publ Pan Am Soc Clin Virol. 2003 Jul;27(2):136–45.

12. Rousseau R, Vanwambeke SO, Boland C, Mori M. The isolation of culturable bacteria in *Ixodes ricinus* ticks of a Belgian peri-urban forest uncovers opportunistic bacteria potentially important for public health. Int J Environ Res Public Health. 2021 Nov 19;18(22):12134.

13. Jado I, Oteo JA, Aldámiz M, Gil H, Escudero R, Ibarra V, et al. Rickettsia monacensis and Human Disease, Spain - Volume 13, Number 9—September 2007 - Emerging Infectious Diseases journal - CDC. [cited 2024 Apr 4]; Available from: https://wwwnc.cdc.gov/eid/article/13/9/06-0186_article

14. Coipan EC, Fonville M, Tijsse-Klasen E, van der Giessen JWB, Takken W, Sprong H, et al. Geodemographic analysis of *Borrelia burgdorferi* sensu lato using the 5S–23S rDNA spacer region. Infect Genet Evol. 2013 Jul 1;17:216–22.

15. Casati S, Sager H, Gern L, Piffaretti JC. Presence of potentially pathogenic *Babesia* sp. for human in *Ixodes ricinus*i n Switzerland. Ann Agric Environ Med. 2017 May 11;13(1):65–70.

**Table S2.** Summary of mix components and run cycles

| Mix components | All pathogen qPCR (except TBEV) | Ixodes/Dermacentor qPCR | TBEV PCR | Babesia conventional PCR | Borrelia conventional PCR | Rickettsia conventional PCR |
| --- | --- | --- | --- | --- | --- | --- |
|  | 10µl Mix (iQ multiplex Powermix -1725848, Bio-Rad, Hercules, USA-), 0,4µl primers and probes*(10µM), 5µl sample, 20µl final volume.  *0,2µl of primes for *Borrelia* FlaB reverse primers and 0,2µl for Borrelia OspA probe | 6,25µl Mix (SYBR Green I Master-04707516001, Roche Diagnostics Nederland B.V, Almere, the Netherlands-), 1µl primers (10µM), 2µl sample, 25µl final volume. | 12,5µl RT-PCR buffer and 1µl RT-PCR enzyme mix (AgPath-ID™ One-Step RT-PCR Reagents-AM1005, Applied Biosystems, Waltham, USA-), 1µl primers and probe (10µM), 8,5µl of sample, 25µl final volume. | 12,5µl HotStart Mix (203443, Qiagen, Aarhus, Denmark), 0.75 µL primers (10µM), 3µl of sample, 25µl final volume. | 12,5µl HotStart Mix (203443, Qiagen, Aarhus, Denmark), 1µL primers (10µM), 3µl of sample, 25µl final volume. | 2µL buffer and 1.6µl dNTPs and 0.6µl MgCl2 and 0.2µl Taq (Taq DNA polymerase recombinant kit-InvitrogenTM, Thermo Fisher Scientific, Waltham, MA, USA-), 0.2µL primers (20µM), 5µL sample, 22µl final volume. |
| PCR conditions |  |  |  |  |  |  |
| Reverse transcription | / | / | 50°C, 15min (1cycle) | / | / | / |
| Step 1 | 95°C, 5min (1cycle) | 95°C, 10min (1cycle) | 95°C, 10min (1cycle) | 94°C, 15min (1cycle) | 94°C, 5min (1cycle) | 94°C, 15min (1cycle) |
| Step 2 | 94°C, 5sec, 60°C, 35sec (45cycles) | 95°C, 8sec, 60°C, 5sec, 72°C, 5sec (50cycles) | 95°C, 15sec, 60°C, 35 sec (45 cycles) | 94°C, 20sec, 67°C, 30sec, 72°C, 30sec (10cyles lowering 1°C per cycle) | 94°C, 20sec, 70°C, 30sec, 72°C, 30sec (10cyles lowering 1°C per cycle) | 95°C, 20sec, 48°C, 30sec, 60°C 2min (35cycles) |
| Step 3 | / | 65°C, 1sec, 95°C continuous | / | 94°C, 20sec, 57°C, 30sec, 72°C, 30sec (40cycles) | 94°C, 20sec, 60°C, 30sec, 72°C, 30sec (40cycles) | 72°C, 10min |
| Step 4 | / | / | / | 72°C, 7min (1cycle) | 72°C, 7min (1cycle) | / |
