# Supplementary material for: The prevalence of pathogens in ticks collected from humans in Belgium, 2021, versus 2017
Source: Parasit Vectors. 2024 Sep 5;17:380. doi: 10.1186/s13071-024-06427-x (PMC11378490; doi:10.1186/s13071-024-06427-x)
Supplement: Supplementary file 2 — Additional file 2. Analysis of 2021 prevalences by characteristics and co-infections. Table S3. Pathogen prevalence in feeding ticks on humans in 2021, according to demographics and other characteristics. Table S4. Number of ticks co-infected by pathogen in 2021. [file 13071_2024_6427_MOESM2_ESM.docx]

**Additional file 2: Analysis of 2021 prevalences by characteristics and co-infections**

**Table S3.** Pathogen prevalence in feeding ticks on humans in 2021, according to demographics and other characteristics

|  | *B. burgdorferi* (s.l.)  % pos (95% CI) | *A. phagocytophilum*  % pos (95% CI) | *B. miyamotoi*  % pos (95% CI) | *N. mikurensis*  % pos (95% CI) | *Babesia* spp.  % pos (95% CI) | *R. helvetica*  % pos (95% CI) |
| --- | --- | --- | --- | --- | --- | --- |
| Age | p=0.295 | p=0.435 | p=0.682 | p=0.400 | p=0.158 | p=0.077 |
| <15 (n=142) | 9.9 (5.9-16) | 2.8 (1.1-7.3) | 2.8 (1.1-7.3) | 1.4 (0.4-5.5) | 0 (0-2.6) | 10.6 (6.5-16.8) |
| 15-24 (n=34) | 2.9 (0.4-18.1) | 5.9 (1.5-20.7) | 0 (0-10.3) | 0 (0-10.3) | 0 (0-10.3) | 17.6 (8.1-34.1) |
| 25-44 (n=181) | 9.4 (5.9-14.6) | 3.3 (1.5-7.2) | 1.7 (0.5-5) | 4.4 (2.2-8.6) | 2.8 (1.2-6.5) | 18.2 (13.3-24.5) |
| 45-64 (n=249) | 13.3 (9.6-18.1) | 5.2 (3.1-8.8) | 2.8 (1.3-5.8) | 4 (2.2-7.3) | 0.8 (0.2-3.2) | 9.6 (6.5-14) |
| 65+ (n=286) | 8.7 (6-12.6) | 6.3 (4-9.8) | 3.8 (2.1-6.8) | 2.4 (1.2-5) | 2.4 (1.2-5) | 14.3 (10.7-18.9) |
| Region | p=0.854 | p=1 | p=1 | p=0.763 | p=0.817 | p=0.279 |
| Brussels (n=8) | 12.5 (1.7-53.7) | 0 (0-36.9) | 0 (0-36.9) | 0 (0-36.9) | 0 (0-36.9) | 12.5 (1.7-53.7) |
| Flanders (n=477) | 10.1 (7.7-13.1) | 4.6 (3.1-6.9) | 2.9 (1.7-4.9) | 3.4 (2.1-5.4) | 1.5 (0.7-3) | 14.7 (11.8-18.1) |
| Wallonia (n=406) | 10.1 (7.5-13.4) | 4.7 (3-7.2) | 2.7 (1.5-4.8) | 2.7 (1.5-4.8) | 1.7 (0.8-3.6) | 11.3 (8.6-14.8) |
| Period (months) | p=0.854 | P=0.151 | P=0.588 | p=0.619 | p=0.274 | p=0.572 |
| April-June (n=605) | 10.2 (8.1-12.9) | 5.8 (4.2-8) | 3.3 (2.1-5.1) | 3 (1.9-4.7) | 1.7 (0.9-3) | 14 (11.5-17.1) |
| July-August (n=255) | 9 (6.1-13.2) | 2.7 (1.3-5.6) | 2 (0.8-4.6) | 2.4 (1.1-5.1) | 0.8 (0.2-3.1) | 12.2 (8.7-16.8) |
| September-October (n=68) | 10.3 (5-20.1) | 2.9 (0.7-11) | 2.9 (0.7-11) | 4.4 (1.4-12.8) | 2.9 (0.7-11) | 10.3 (5-20.1) |
| Type of environment | p=0.951 | p=0.888 | p=0.769 | p=0.718 | p=0.045* | p=0.049* |
| Wood/forest (n=263) | 8.7 (5.9-12.8) | 4.2 (2.3-7.4) | 2.7 (1.3-5.5) | 3.4 (1.8-6.4) | 3.4 (1.8-6.4)^a^ | 14.4 (10.7-19.2) |
| Garden (n=409) | 11 (8.3-14.4) | 5.4 (3.6-8) | 3.7 (2.2-6) | 2.4 (1.3-4.5) | 0.7 (0.2-2.2)^a^ | 10.5 (7.9-13.9) ^b^ |
| Nature reserve, not forest (n=71) | 11.3 (5.7-20.9) | 7 (3-15.8) | 1.4 (0.2-9.3) | 5.6 (2.1-14.1) | 0 (0-5.1) | 21.1 (13.2-32.1) ^b^ |
| Grassland, agricultural field (n=48) | 10.4 (4.4-22.7) | 4.2 (1-15.2) | 0 (0-7.4) | 2.1 (0.3-13.4) | 4.2 (1-15.2) | 22.9 (13.2-36.8) ^b^ |
| Other (n=35) | 8.6 (2.8-23.4) | 2.9 (0.4-17.7) | 0 (0-10) | 2.9 (0.4-17.7) | 0 (0-10) | 8.6 (2.8-23.4) |
| Unknown (n=66) | 9.1 (4.1-18.8) | 3.0 (0.8-11.3) | 3.0 (0.8-11.3) | 3.0 (0.8-11.3) | 0 (0-5.4) | 13.6 (7.3-24.2) |
| Activity of person bitten | p=0.624 | p=0.435 | p=0.572 | p=0.343 | p=0.865 | p=0.006* |
| Leisure (n=716) | 10.5 (8.4-12.9) | 5.2 (3.8-7.1) | 2.7 (1.7-4.1) | 2.9 (1.9-4.5) | 1.7 (1-2.9) | 13.8 (11.5-16.6) **^c^** |
| Professional (n=34) | 2.9 (0.4-18.1) | 8.8 (2.9-24) | 2.9 (0.4-18.1) | 0 (0-10.3) | 0 (0-10.3) | 23.5 (12.2-40.5) **^c^** |
| Other (n=86) | 10.5 (5.5-18.9) | 2.3 (0.6-8.8) | 4.7 (1.8-11.7) | 5.8 (2.4-13.2) | 0 (0-4.2) | 3.5 (1.1-10.3)**^c^** |
| Unknown (n=36) | 8.3 (2.7-22.9) | 2.8 (0.4-17.3) | 2.8 (0.4-17.3) | 2.8 (0.4-17.3) | 0 (0-9.7) | 11.1 (4.2-26.1) |

^a^ Significantly lower in ‘garden’ compared to ‘wood/forest’ (p=0.015).

**^b^** Significantly lower in ‘garden’ compared to ‘nature reserve, not forest’ (p=0.017) and compared to ‘grassland, agricultural field’ (p=0.018).

^c^ Significantly lower in category’ other’ compared to ‘professional’ (p=0.002) and compared to ‘leisure’ (p=0.003).

* Significant p-values

**Table S4.** Number of ticks co-infected by pathogen in 2021

|  | N co-infected /  N infected (%) | *B. burgdorferi (*s.l.) | *A. phagocytophilum* | *B. miyamotoi* | *N. mikurensis* | *Babesia* spp. | *R. helvetica* |
| --- | --- | --- | --- | --- | --- | --- | --- |
| *B. burgdorferi (*s.l.) | 32/92 (34.8%) | - | 5 | 4 | 10*** | 4* | 12 |
| *A. phagocytophilum* | 13/44 (29.5%) | 5 | - | 0 | 1 | 0 | 9 |
| *B. miyamotoi* | 5/27 (18.5%) | 4 | 0 | - | 0 | 0 | 2 |
| *N. mikurensis* | 13/27 (48.1%) | 10*** | 1 | 0 | - | 0 | 2 |
| *Babesia* spp. | 8/14 (57.1%) | 4* | 0 | 0 | 0 | - | 4 |
| *R. helvetica* | 26/123 (21.1%) | 12 | 9 | 2 | 2 | 4 | - |

*p<0.05; **p<0.01; ***p<0.001
