# Supplementary material for: The prevalence of pathogens in ticks collected from humans in Belgium, 2021, versus 2017
Source: Parasit Vectors. 2024 Sep 5;17:380. doi: 10.1186/s13071-024-06427-x (PMC11378490; doi:10.1186/s13071-024-06427-x)
Supplement: Supplementary file 3 — Additional file 3. Results for Spiroplasma ixodetis, Francisella tularensis and Coxiella burnetii. Table S5. Pathogen prevalence in feeding ticks on humans in 2017 (1225 nymphs and 290 adults) and 2021 (792 nymphs and 136 adults) by life stage. Table S6. Prevalence of Spiroplasma ixodetis, Francisella tularensis and Coxiella burnetii in feeding ticks on humans in 2021, according to demographics and other characteristics. [file 13071_2024_6427_MOESM3_ESM.docx]

**Additional file 3: Results for *Spiroplasma ixodetis*, *Francisella tularensis* and *Coxiella burnetii***

**Table S5.** Pathogen prevalence in feeding ticks on humans in 2017 (1,225 nymphs and 290 adults) and 2021 (792 nymphs and 136 adults) by life stage

| Pathogen | Tick life stage | 2017 | 2021 | p-value 2017 vs. 2021 |
| --- | --- | --- | --- | --- |
| *S. ixodetis* | Nymphs | 13.1% (11.4-15.2) | 10.5% (8.5-12.8)^a^ | 0.073 |
|  | Adults | 14.8% (11.2-19.4) | 22.1% (15.9-29.8) ^a^ | 0.065 |
|  | Total | 13.5% (11.8-15.3) | 12.2% (10.2-14.4) | 0.358 |
| *F. tularensis* | Nymphs | NA | 0.1% (0-0.9) | NA |
|  | Adults | NA | 0% (0-2.7) | NA |
|  | Total | NA | 0.1% (0-0.8) | NA |
| *C. burnetii* | Nymphs | NA | 0.6% (0.3-1.5) | NA |
|  | Adults | NA | 0% (0-2.7) | NA |
|  | Total | NA | 0.5% (0.2-1.3) | NA |

^a^ Statistically significant different in adults compared to nymphs within the same year (P<0.001)

One out of eight *I. hexagonus* ticks (nymph) and four larvae were infected with *S. ixodetis* in 2021.

**Table S6.** Prevalence of *S. ixodetis*, *F. tularensis and* *C. burnetii* in feeding ticks on humans in 2021, according to demographics and other characteristics

|  | *S. ixodetis*  % pos (95% CI) | *F. tularensis*  % pos (95% CI) | *C. burnetii*  % pos (95% CI) |
| --- | --- | --- | --- |
| Age | P=0.592 | P=0.679 | P=0.735 |
| <15 (n=142) | 15.5 (10.4-22.4) | 0 (0-2.6) | 0.7 (0.1-4.8) |
| 15-24 (n=34) | 14.7 (6.3-30.8) | 0 (0-10.3) | 0 (0-10.3) |
| 25-44 (n=181) | 11 (7.2-16.5) | 0 (0-2) | 1.1 (0.3-4.3) |
| 45-64 (n=249) | 10.4 (7.2-14.9) | 0.4 (0.1-2.8) | 0.4 (0.1-2.8) |
| 65+ (n=286) | 13.3 (9.8-17.7) | 0 (0-1.3) | 0.3 (0-2.4) |
| Region | P<0.0001* | P=0.465 | P=1 |
| Brussels (n=8) | 0 (0-36.9) | 0 (0-36.9) | 0 (0-36.9) |
| Flanders (n=477) | 17.6 (14.4-21.3)^a^ | 0 (0-0.8) | 0.6 (0.2-1.9) |
| Wallonia (n=406) | 6.2 (4.2-9)^a^ | 0.2 (0-1.7) | 0.5 (0.1-1.9) |
| Season | P=0.883 | P=0.348 | P=1 |
| April-June (n=605) | 12.6 (10.1-15.4) | 0 (0-0.6) | 0.7 (0.2-1.7) |
| July-August (n=255) | 11.4 (8-15.9) | 0.4 (0.1-2.7) | 0.4 (0.1-2.7) |
| September-October (n=68) | 11.8 (6-21.8) | 0 (0-5.3) | 0 (0-5.3) |
| Type of environment | P=0.692 | P=0.559 | P=0.478 |
| Wood/Forest (n=263) | 14.1 (10.4-18.8) | 0.4 (0.1-2.6) | 0.8 (0.2-3) |
| Garden (n=409) | 11 (8.3-14.4) | 0 (0-0.9) | 0.2 (0-1.7) |
| Nature reserve, not forest (n=71) | 11.3 (5.7-20.9) | 0 (0-5.1) | 1.4 (0.2-9.3) |
| Grassland, agricultural field (n=48) | 16.7 (8.6-29.9) | 0 (0-7.4) | 0 (0-7.4) |
| Other (n=35) | 14.3 (6.1-30) | 0 (0-10) | 0 (0-10) |
| Unknown (n=102) | 9.8 (5.4-17.3) | 0 (0-3.6) | 1 (0.1-6.6) |
| Activity of person bitten | P=0.419 | P=1 | P=0.727 |
| Leisure (n=716) | 13.1 (10.8-15.8) | 0.1 (0-1) | 0.6 (0.2-1.5) |
| Professional (n=34) | 11.8 (4.5-27.5) | 0 (0-10.3) | 0 (0-10.3) |
| Other (n=86) | 9.3 (4.7-17.5) | 0 (0-4.2) | 0 (0-4.2) |
| Unknown (n=92) | 7.6 (3.7-15.1) | 0 (0-3.9) | 1.1 (0.2-7.3) |

^a^ Statistically significant different between Flanders and Wallonia (P<0.0001)

* Significant p-value
